# Education-based grant programmes for bottom-up distance learning and project catalysis: antimicrobial resistance in Sub-Saharan Africa

**DOI:** 10.1099/acmi.0.000472.v3

**Published:** 2023-03-15

**Authors:** Chris L. B. Graham, Harry Akligoh, Joy King Ori, Gameli Adzaho, Linda Salekwa, Patrick Campbell, Courage K. S. Saba, Thomas E. Landrain, Marc Santolini

**Affiliations:** ^1^​ Just One Giant Lab, Paris, France; ^2^​ University of Warwick, Coventry, UK; ^3^​ Duplex Bioscience LBG, Accra, Ghana; ^4^​ Mbeya University of Science and Technology, Mbeya, Tanzania; ^5^​ University for Development Studies, Tamale, Ghana; ^6^​ Université Paris Cité, INSERM, U1284, F-75004 Paris, France

**Keywords:** AMR, community science, curricula, funding, grant review, training

## Abstract

International development and aid are often conducted through the allocation of funding determined by decisions of non-locals, especially in the west for those in the global south. In addition, such funding is often disassociated from local expertise, therefore providing little long-term developmental impact and generating distrust. This is particularly true for conservation, as well as environmental and educational programmes. We hypothesize that by granting local people the educational tools and the necessary funding to develop their own projects through the use of an applicant-driven peer-review approach, it is possible to relocalize the decision-making process to the programme participants, with the potential to generate and select more relevant projects with developmental outcomes of higher quality. Here we created an online curriculum for antimicrobial resistance (AMR) education that was followed by 89 participants across Ghana, Tanzania, Nigeria and Uganda. We then created an open research programme that facilitated the creation of eight *de novo* projects on AMR. Finally, we organized an applicant-driven grant round to allocate funding to the ‘Neonatal Sepsis in Nigeria’ project to conduct a pilot study and awareness campaign. This work opens perspectives for the design of frugal educational programmes and the funding of context-specific, community-driven projects aimed at empowering local stakeholders in the global South.

## Data Summary

Supplementary Material files can be retrieved using the following link on Figshare: https://doi.org/10.6084/m9.figshare.20418828.v2 [1]. This includes a pdf of the form used for applicant selection, supplementary information and method notes, the reviewer form .pdf file and the location data determined by Microbis [2] and Geocoder. Included in this Figshare is Fig. S1, which summarizes this data.

## Introduction

### Antimicrobial resistance (AMR)

Harmful bacteria cause more deaths per year worldwide than either AIDs or Malaria [3]. For most of these bacteria, effective antibiotic treatments are available. This has been true since the advent of penicillin, and the following decades uncovered many more antimicrobials capable of saving human life. However, globally bacteria have become resistant to many of our most effective and widely available antibiotics. This resistance is not yet adequately studied or monitored, with problem areas in the global south especially comprising the overuse of antibiotics in food crops and livestock, and their overspill into water sources and effect the local environment and peoples, and allowing for spread of resistance, constituting a *One Health* problem. Natural selection of antibiotic-resistant strains in these antibiotic-rich environments gives an advantage to new and emerging resistant strains in rural and urban settings globally, as well as clinical settings where antibiotics are more commonly used and required for medical treatment [4]. Soon this will inevitably lead to an antibiotic shortage.

### LMIC contextualized by AMR

Low and middle-income countries (LMICs) bear the highest burdens of infectious diseases with potentially the least resources, and also have limited data on the epidemiology and burden of antimicrobial resistance [5, 6]. A 2017 WHO report highlighted the gaps in information on pathogens of major public health threats. The report also emphasized the lack of high-quality data and how it limits our ability to assess and monitor trends of resistance worldwide [7]. Congruent to earlier reports, the laboratory identification of AMR and bacteria still rely on the use of substandard phenotypic techniques coupled with poor laboratory information management systems seen mostly in LMICs such as within Africa. Aside from this, other barriers such as a poor diagnostic infrastructure, limited staff capacity and training as well as questionable quality-management systems compound the problems of tracking down resistance [8, 9].

The COVID-19 pandemic has underscored the importance of new paradigms for testing and researching infectious pathogens. The use of pathogen genomics has provided information to scientists in record time to help guide public health strategies. Hence recent efforts in Nigeria saw researchers use sequencing to study and characterize sequence types (STs) for AMR bacteria causing hospital-acquired infections [10]. Datasets collected were made available to their AMR National Coordinating Centre (NCC) in an effort towards overcoming the current knowledge and fragmented data gap. Gains made by countries in Europe in the fight against antimicrobial resistance have largely been due to the availability of data at several levels and education, which is mostly absent in LMICs [11]. Therefore, in the face of growing global health threats, where individualism is a danger to gains made, LMICs particularly those in Africa are optimistic about initiatives that aid their developmental agenda in strengthening existing but weak or non-existent infrastructure to tackle health threats like AMR.

### Education and grassroots project creation programmes

There have been efforts in the past to improve the infrastructure of LMICs, especially in Africa, however the majority of these programmes have been initiated by projects coming from a western nation with control over project management at all levels [12]. This has recently begun to change, with an emphasis towards projects that give natives project ownership and motivation, creating sustainable projects without outside interference.

Therefore, similar to this emphasis on bottom-up – as opposed to top-down – change, a concept of ‘smart people before smart cities’ has become dominating sentiment, with the creation of opportunities for residents, in particular educational ones, being a priority that must go hand in hand with infrastructure improvements [13]. This capacity building in people and facilitation of independent work therefore must take priority and allow for new decentralized teams on the continent to work on global problems using their own motivation and methods. This has been shown to be more effective at fostering long-term change [14].

### Online learning and capacity building are a priority over travel

During the COVID-19 pandemic, international programmes of educational support were encouraged to make educational resources available online and disseminated locally where possible. This has shown to work well in some settings such as high schools [14, 15] as well as within new online infrastructure, such as the open source and African built ‘Voltschool’ [16]. Similarly the funding granted for these international programmes can be more efficiently spent on capacity building rather than on flights. Prior to this study, the researchers conducted another study focusing on an agile funding allocation scheme to projects determined by applicants, and found that the correlation between reviews, with reviewers as applicants was similar to non-applicant reviewers in other schemes, and of high efficiency [17]. Following this scheme, here we used the travel spending funds towards a microgrant allocation to allow the applicants within our curriculum cohort to determine a project within their cohort that should receive seed funding for capacity building beyond education, as well as motivate their own ideas with *de novo* project creation.

### Goal of study

Just One Giant Lab (JOGL) is an NGO for collaborative science. In partnership with Hive Biolab, Kumasi, Ghana, University for Development Studies (UDS), Tamale, Ghana and Mbeya University of Science and Technology (MUST) Tanzania, and with the support from the Microbiology Society, JOGL hosted a grant round to catalyse project creation by participants in response to the need for increased capacity building and a focus on education organized a virtual training series on antimicrobial resistance (AMR). We also provided a training on the use of an AMR tracking app ‘microBIS.io’ for a pilot result. The goal of the workshop was to build the capacity of university students and laboratory technicians on the latest trends in AMR screening and testing, as well as increase AMR stewardship and empower them with the resources to become agents of change in reversing the spread and negative effects of AMR in Africa.

## Methods

All participants in the AMR workshop were invited to join the ‘Africa against AMR Community’ space on the JOGL collaborative network platform [18, 19] as well as a special Africa AMR channel within a community workspace on instant messaging service ‘Slack’ [20] to enhance networking and collaboration.

**Fig. 1.**
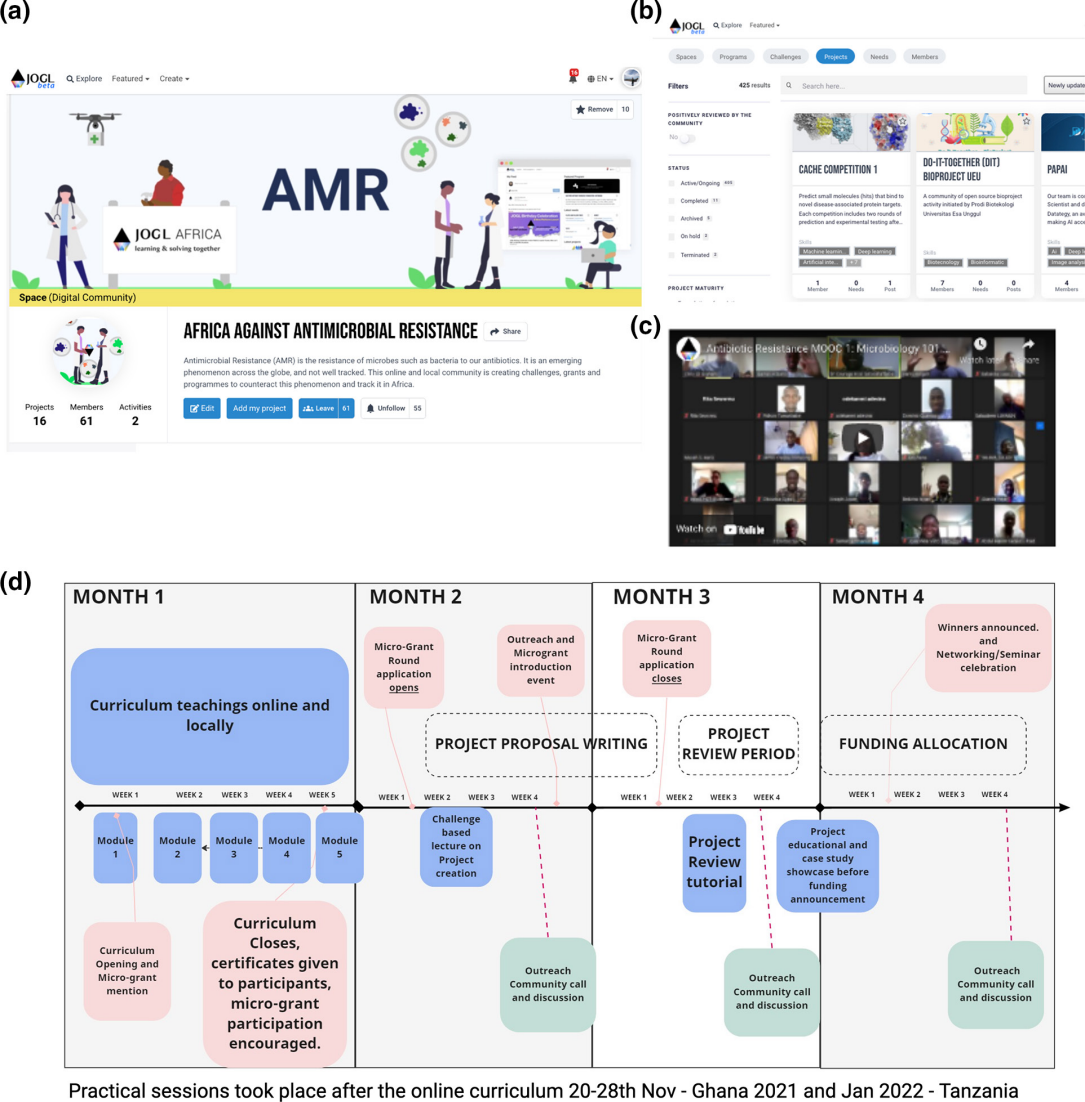
Online project creation and networking. (a) Programme page in the JOGL social innovation network used to allow for project creation in an open-source accessible website. (b) Project search and display function. (c) Videos of modules were recorded and displayed on the platform. (d) Timeline of initiative: pink – microgrant related deadlines; green/light green – community call; blue – curriculum and lectures.

The project team developed a curriculum to cover important aspects of microbiology, AMR, and innovative approaches to tackle the challenge Finally, at the end of the programme a grant round was conducted. In person practical sessions were held separately from the main programme, to coordinate with term times for participating academics, in Tanzania and Ghana, asynchronously (Fig. 1).

### Geotagging and surveying

Geotags of the coarse-grained information (town level) participant locations were pulled from the JOGL website and converted using Geo-Coder [18, 21]**,** and the names and identifiable aspects of members were removed before geotagging. Upon signup to the platform, consent is given for location tracking, in the user terms and conditions.

### Outreach and recruitment of participants

Participants were contacted through social media channels such as Twitter, Facebook and LinkedIn to advertise a free curriculum and grant round, in addition to on our constructed site. Existing media accounts with 4000+ followers allowed for sharing between individuals through connections.

### Curriculum

The course was composed of two parts – an online workshop series and onsite practical sessions. Each lecture was created 1 week ahead of schedule. The online workshop consisted of five modules delivered as weekly Zoom webinars from 21 September to 19 October. These sessions were recorded and the videos were uploaded on YouTube [22]. Practical sessions on microbial identification and sensitivity testing [23] were scheduled for interested participants in Kumasi, Ghana (Hive Biolab), Ghana, and Mbeya, Tanzania (MUST). The details of the curriculum can be found in the Supplementary Material, available in the online version of this article. No formative assessment was made, however attendance was kept each session through a form.

### Online web-space

For presenting the modules within the ‘Massive Open Online Course’ (MOOC), and the grant review round, we used a citizen science and project creation network called ‘Just One Giant Lab’ [18]. This allowed participants to create their own projects in addition to taking part in our syllabus available here [22]. The social network aspect in a focused programme allowed for sharing and openly disseminating the curriculum internationally. These were all created with the help of the design team at JOGL.

### Local collaborator selection

Coordinators of the programme local to Ghana and Tanzania were found through the JPIAMR portal for collaborator finding, as well as through existing shared contacts. They were reached out through email and the collaboration was initiated at the grant writing stage, with further participants and partners invited after the grant award by our funders.

### Administration

Administration was conducted first through a google form (Data Summary) for the collection of emails. The resulting email spreadsheet, which included demographic information, was then used to liaise for the rest of the course.

**Fig. 2.**
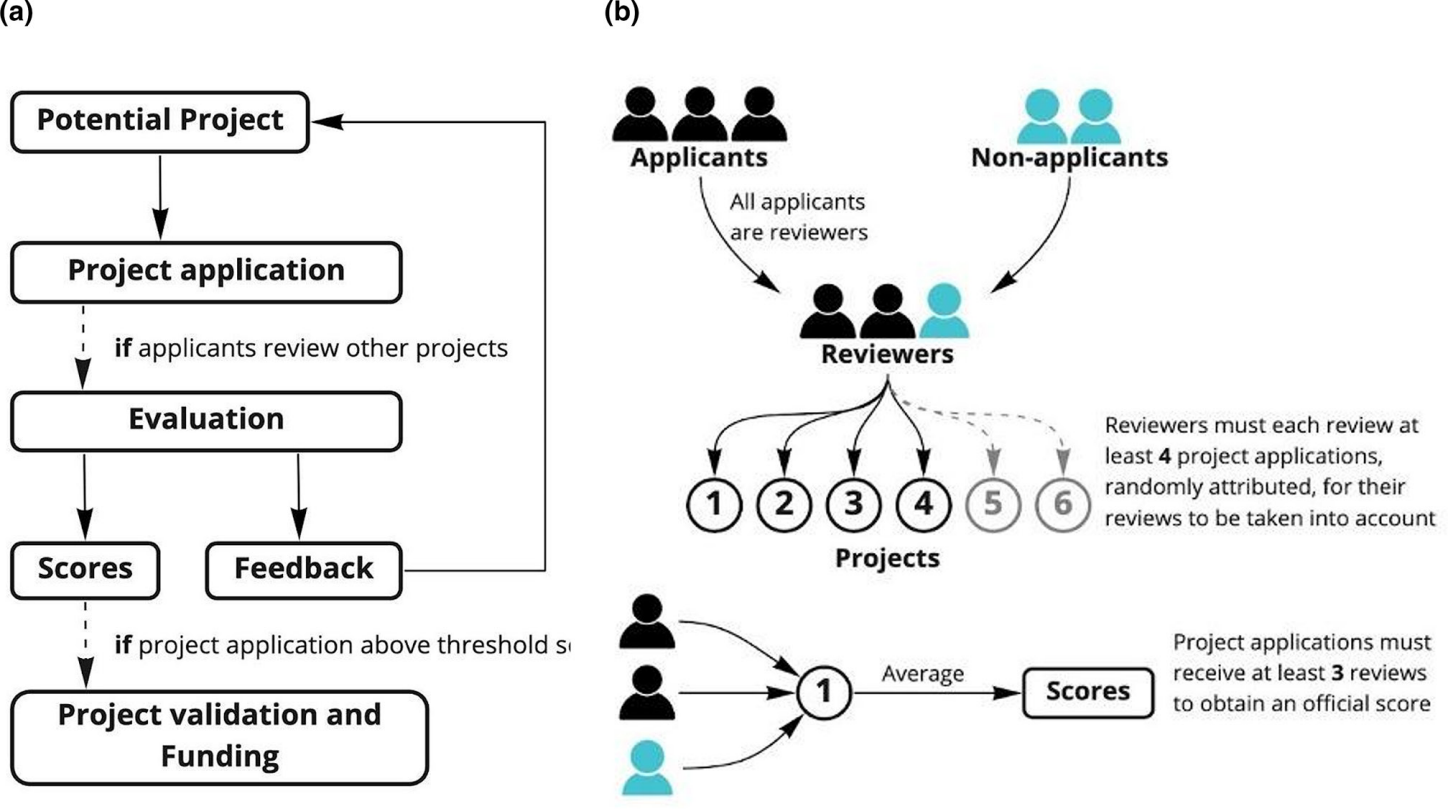
Grant application process. (a) Flow of community review and grant application. (b) Applicants review one another allowing full scalability. (Adapted from Graham, Landrain and Santolini *et a*. 2022) [17].

The grant round (Fig. 2) leveraged the JOGL interface to facilitate contact with applicants. Using the JOGL application programming interface (API), applicant data was collected so that anyone who joined the grant round were then emailed with any updates through the course of the endeavour. Administration within the consortium of collaborators between MUST, University of Warwick, Hive Biolab and JOGL was conducted over Slack channels, emails and Zoom meetings with no in-person meeting. There was one public slack channel, a private administrative channel, and a questions and answers channel. Participants were invited to the programme through social media posts, word of mouth, and by emails to admins of universities and makerspace laboratories in the region, in a campaign that began 2 months prior to the beginning of the course, and involved a sign-up process.

### Network of skills creation

**Fig. 3.**
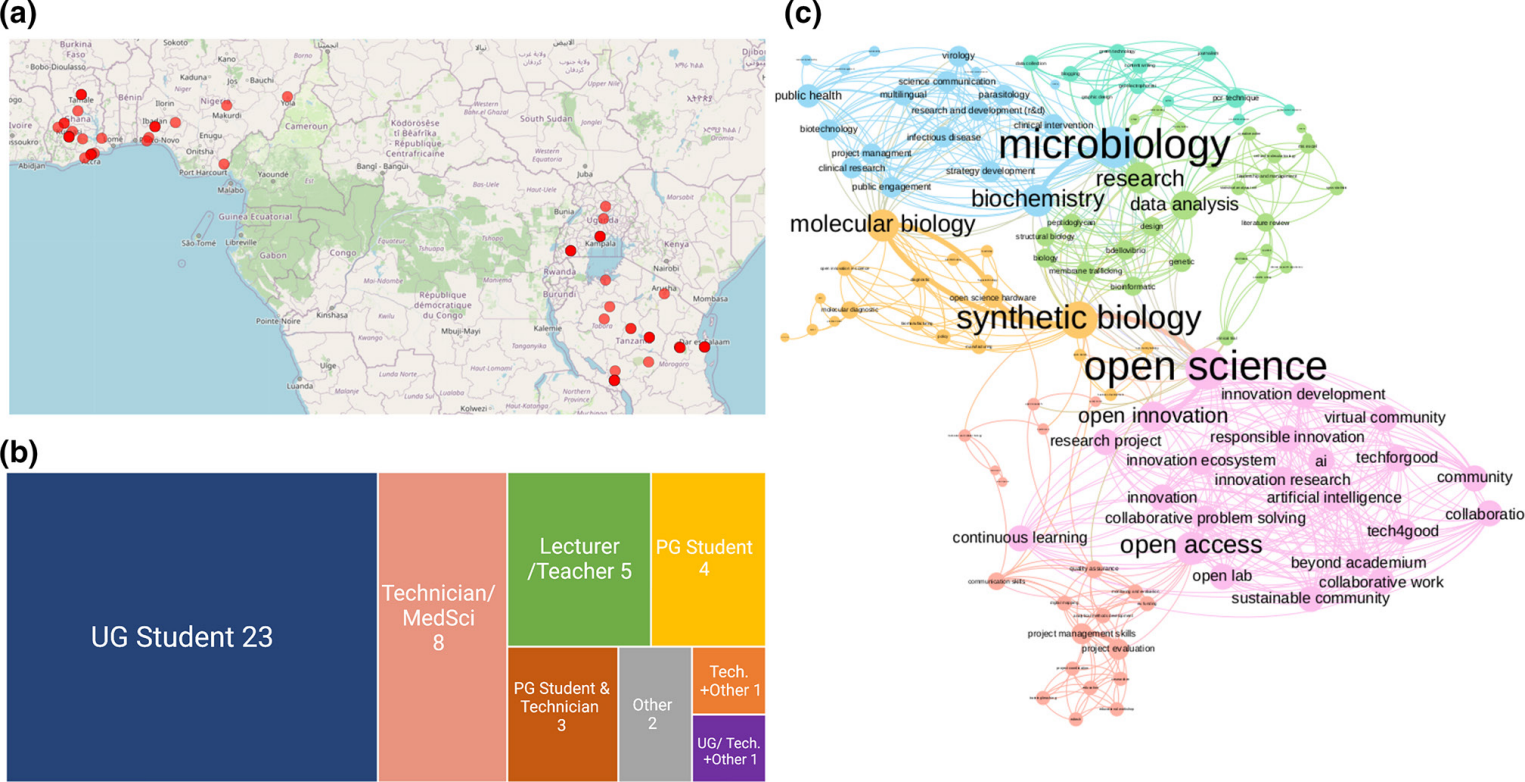
Location and profession of participants of virtual curriculum in Africa. (a) Locations derived from geocoded data from form input. All participants are shown as red dots by city. (b) A visualization of the professions who filled in the post-curriculum form. (c) Network of skills in the cohort of participants, taken information given upon signup to the JOGL platform. Skills are connected if a participant has both of them in their profile. The thickness of connections indicates number of participants sharing two skills and the size of nodes indicates the number of neighbouring nodes. Gephi 0.9.3(22) was used to represent the network, as described in Methods. Colour indicates the community, assessed using modularity.

Similar to Masselot *et al*. [24], participants filled in their professional background, skills and employment status on signing up to the JOGL platform. In order to better understand how skills were related across participants, we used a network approach to assess similarity between skills and got further insights about the global diversity of the community. In this network approach, each declared skill is a node and the considered skills as linked if they co-occur in a participant. Links are then weighted by the number of participants they co-occur in. Gephi 0.9.3 was used to represent the network in Fig. 3 [25], its modularity algorithm was used with default parameters to compute communities.

### AMR in africa grant round

JOGL organized the ‘AMR in Africa Grant Round’ to support one research, innovation or education project tackling AMR in Africa. The call for proposals ran for 1 month, from 18 November to 18 December 2021. We used a proposal template as attached (Data Summary) with a focus on the antimicrobial resistance outcomes to encourage a formal application approach, then used the following format for administration (Fig. 2). Applicants were required to mark at least four other applicants, therefore giving a democratic and local outlook on the most relevant and innovative solutions by curricula through experts and existing creators. This followed a community review methodology as used in the OpenCOVID19 programme 2020-2021 [26].

## Results

### Background of participants and trainers

The virtual training attracted 89 participants (whom attended at least two online sessions) across the African continent from diverse backgrounds; such as laboratory technicians, students, researchers and innovators (Fig. 3). More than 50 % of the participants were undergraduate students, while the other 50 % comprised lab technicians/scientists, postgraduate students, and teachers/lecturers also. Most of the participants came from Ghana, Tanzania, Nigeria and Uganda as per the details filled in the application form. The results of the form indicated our shared connections were greatest in Ghana, Nigeria, Uganda and Tanzania as visualized in Fig. 3. This is likely as our collaborating partners were from Tanzania and Ghana, who also shared the opportunity within their networks, notably from a range of cities across the aforementioned countries (Fig. 3a). The distance between participants, normally inhibitory to practical or in-person instruction, was overcome using online teaching and community building. The community leveraged through the creation of a curriculum was that of a network of participants with some relevant skills already (Fig. 3c) who could complement each other’s strengths during project creation: those with more experience with open science in general, creating a community of open science advocates (including JOGL staff) (pink), but also project developers (orange) and biologists (green and blue), with some having multiple of these skills, indicated by degree of connection between groups. Most notably synthetic biologists who joined the grant and education programme had the most experience in open science.

### Participants engagement and post-MOOC thoughts

**Fig. 4.**
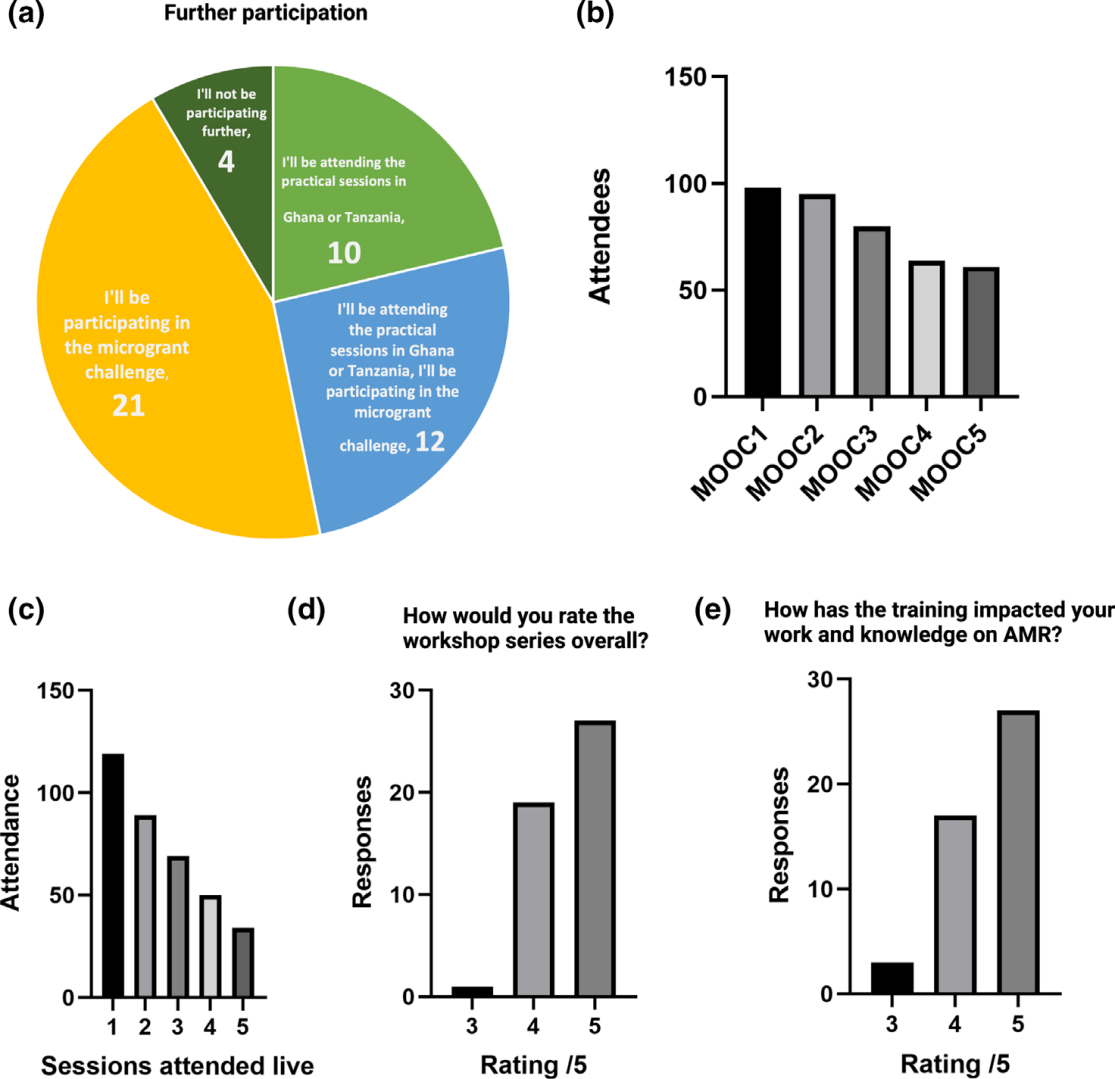
Participant feedback of pre-grant curriculum. (a) Participation, practically, in grant challenge or no further participation. (b) Attendance of online MOOC lectures. (c) Attendance of live lectures. (d) Rating of curriculum [3–7], according to participant feedback. (e) Impact of programme on future work [3–7].

Whilst 98 attendees attended the first online seminar, this over-time reduced to 61 participants by MOOC5 who could attend the seminars live, with the recordings later available on YouTube, however 119, 89, 69, 50 and 34 of the attendees attended at least one, two, three, four and five of the sessions live (Fig. 4c), respectively, suggesting attendees joined various lectures live depending on relevance or convenience. Despite the drop in attendees in the Zoom call, the overall feedback shared by the participants in a post-workshop survey was positive (Fig. 4). Roughly 46 of the 47 survey respondents rated the workshop 4 or 5 (1–5 scale) (Fig. 4a). Similarly, over 44 of the respondents indicated that the training had a high positive impact on their current work (ratings of 4 or 5) (Fig. 4d). Of the modules taught during the online sessions, less specialist introductory aspects such as MOOC 2, MOOC 3 and MOOC 1 (Supplementary Material – Methods and notes) were deemed to be of the highest relevance to the participants trained. Importantly, there was a large number of applicants who continued to apply for the grant round in AMR project creation, suggesting curricula are a positive means for *de novo* project creation and onboarding (Fig. 4b).

Some participants shared additional content about their experiences during the five-part online training:

“I had ‘locked’ myself but the course now opened my mind. It woke me up to see the ever increasing challenge of AMR.”

“The training was well organised, precise and straight to the main point.”

“The modules were very relatable to my work and also improved my knowledge on AMR”

“It was lively at all times and encouraged participatory discourse”.

They also made some suggestions for improvement, some of which have been captured below:

“You may please consider a session to educate non-specialists because, I am not working with a medical institution and having difficulties engaging fully”

“Engage more stakeholders for example political policymakers and other African country health ministries”

“Involve practical sessions for participants from other countries outside Ghana and Tanzania”

“I think you are doing a great job but I think what needs to be improved should be the technical aspect of it because we lost connection at a point during one module.’

Participants’ comments on modules they found relevant (related to informational content and not developmental or project-related content) may be due to the fact that the workshop was dominated by undergraduate students and early career lab technicians, who identified concepts they could apply in their day-to-day work. However, the modules on antimicrobial stewardship and digital mapping are equally relevant in developing additional leadership, coordination and digital literacy skills required for the next level of careers. Comments in particular on ministerial and governmental cooperation to improve outcomes and network are particularly significant changes that future programmes could integrate.

In summary, the AMR workshop and curriculum as a means to build new projects were carried out to a satisfactory level with future iterations likely to alter the curriculum based on signup and topic interest, based on the team’s experience organizing and delivering the programme, and the feedback received from the participants (Fig. 4) . There is a great potential to scale the workshop and its associated activities to reach more students, young professionals, and general science enthusiasts in Africa due to its mediation through online platforms.

### Grant and project creation outcomes

**Fig. 5.**
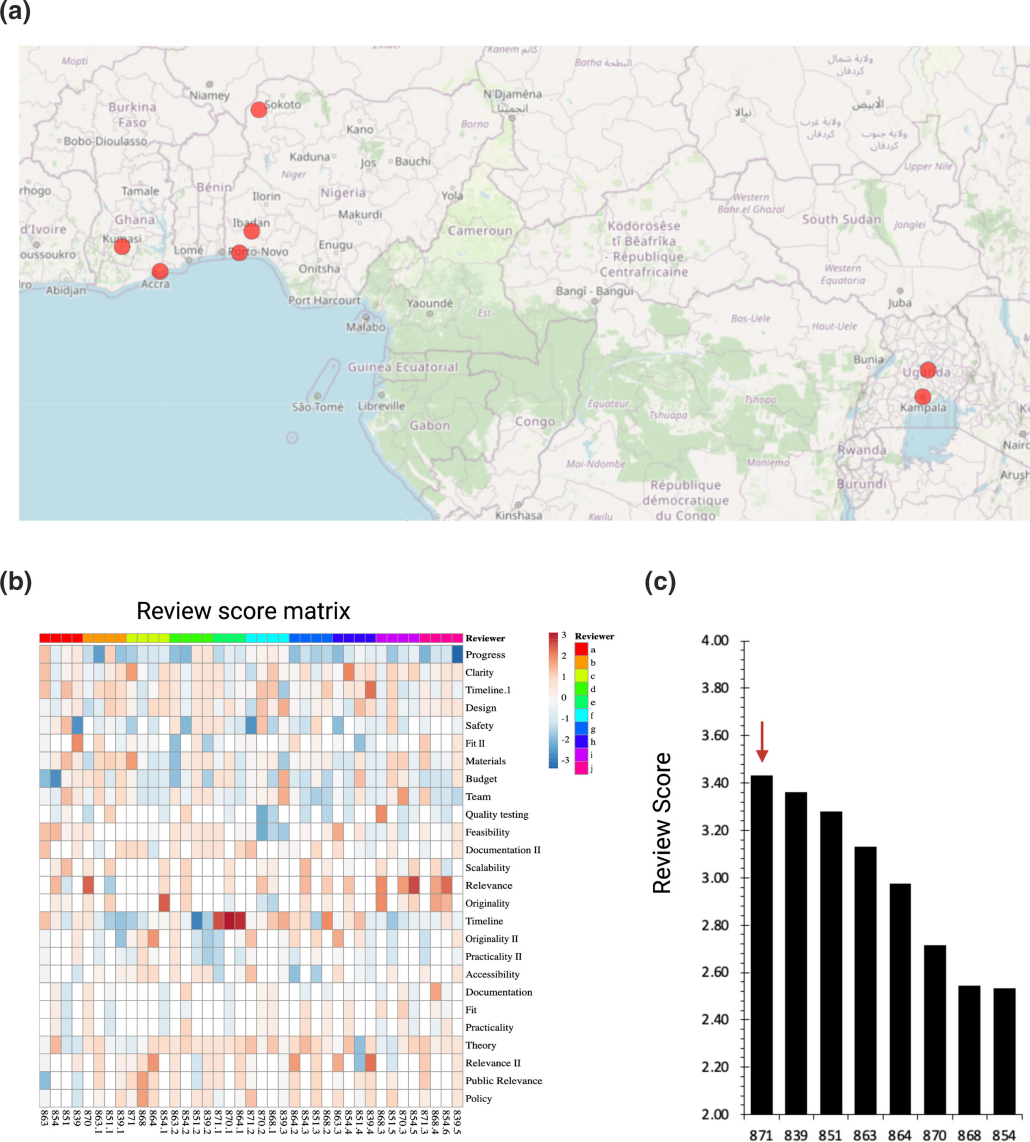
Project catalysis and scoring by applicant-based peer review. (a) Location of new projects created [18, 21], (b) Review behaviour as visualized by heatmap of scores per question red to blue, high scoring to low scoring respectively, determined by Z score, tree of review similarity adjacent. (c) Review score rankings by applicants. Winning project is denoted by a red arrow.

After the curricula, in total eight projects [18] applied to the grant round from a subset of the curriculum takers, but also from individuals who saw the grant round advertised in their network, with a geographical distribution similar to the participant distribution (Fig. 2/Fig. 5a). In order to ensure fairness after submission and review and prevent gamification, the reviewers’ scores across the questions in each form were normalized to their overall average review mark across their five reviews using the community review method. The distribution of scores per question shown by the heatmap indicates independence of questions asked in the form, and allowed for detection of odd reviewer behaviour [17]. (Fig. 5b). The winning team with the highest average review score for the 1000 euro grant was ‘Neonatal Sepsis in Nigeria’ [27]. The other seven teams that applied to the grant, or formed as a consequence of its existence were encouraged to apply to later or other grants. As a result of the programme there is still an active community of collaborators on the community ‘slack’ channels whom conduct meetings on science policy on a monthly basis in preparation for future programmes, and this initiative acted as a catalyst for its community growth, we are currently writing collaborative grants with many of the participants. This shows that with little organization and a small prize pot, a significant collaborative output can be achieved with micro-grants if accompanied by training and encouragement.

### Network building and community science considerations

In addition to the projects created, by creating a community internationally through the web, the collection of information for database-related programmes can be improved. A collaborator who took part in the education aspect of the programme, asked participants working in hospitals to gather data of location, and resistance of bacterial strains, which allowed the team to begin the creation of an antimicrobial resistance location database. Further success in reaching out to participants would allow an international map, and service not possible in on the ground programmes. We have supplied a sample of this data for illustrative purposes (Fig. S1). In future programmes, such communities can be leveraged for similar community science outputs such as data gathering.

### Fostering of collaboration among organizers and participants

Some of the participants met virtually for the first time because of the project. Throughout the development of the courses and practical sessions, there has been exchanges of good practices among participants who are from different countries and continents. The high level of collaboration shown throughout the project paved the way for future joint proposal writing for a bigger grant to curb the menace of AMR worldwide, and an active online science sharing community. Some participants have networked during the workshop and we hope this project provided the right platform for early career scientists in AMR to share their experiences with one another to tackle issues of AMR in Africa.

## Conclusion

Through the use of a local network of contacts and the ‘Just One Giant Lab’ web platform, we were able to create an online curriculum for AMR and deliver this to a diverse audience-based predominantly around Uganda, Nigeria, Tanzania and Ghana. We hosted a programme fostering the creation of eight *de novo* projects and organized a community review round that funded one project selected by applicants and participants of the online curriculum. As such, we performed a hybrid model of online curriculum and grant allocation. We worked with local partners and participants as community drivers, with democratic and local values upheld by decisions of applicants themselves, whilst encouraging a new collaborative project-based perspective in antimicrobrial resistance for medical staff, technicians, students and others.

The techniques of applicant-driven grant decision-making, and online delivery of free educational resources as a pathway for science and awareness funding, provided some success across multiple sub-saharan African nations on a small scale. This method with further funding could help foster development in a cost-efficient manner and at an international scale. This programme and its scale would not have otherwise been possible in person due to travel costs, yet through an online medium, it allowed for capacity building with an educational programme as well as local project creation and curation. This technique opens perspectives to design frugal approaches, allowing a programme team to locally empower individuals in context-specific project catalysis and science education. The authors would encourage other similar initiatives, and also find it likely that if the initiative centred on antimicrobial resistance was possible in areas as culturally diverse as the regions presented it would be replicable in other consortiums nationally and internationally elsewhere, especially the global south.
